# A novel indicator-based visualisation method to investigate diffusion behaviour of dissolved CO_2_ in hydrogels

**DOI:** 10.1016/j.mex.2025.103225

**Published:** 2025-02-17

**Authors:** Laura Fladung, Sarah Vanessa Langwald, Olaf Kruse, Anant Patel

**Affiliations:** a WG Fermentation and Formulation of Biologicals and Chemicals, Faculty of Engineering and Mathematics, Hochschule Bielefeld – University of Applied Sciences and Arts, Interaktion 1, 33619 Bielefeld, Germany; b WG Algae Biotechnology and Bioenergy, Center for Biotechnology, Bielefeld University, Universitätsstraße 25, 33615 Bielefeld, Germany

**Keywords:** Dissolved CO_2_, Diffusion, Porous media, Hydrogels, pH indicator, Non-invasive visualisation, Encapsulation, Immobilisation, Entrapment, Microalgae, Cyanobacteria, Photosynthesis, Indicator-based Dye front Analysis (IDA)

## Abstract

Biocompatible hydrogels are versatile platforms for encapsulating living cells in biotechnology due to their unique physical, structural and mechanical properties. The diffusion of dissolved carbon dioxide (dCO_2_) into the hydrogel matrix is of great importance for the growth of immobilised photosynthetic cells like microalgae and cyanobacteria. However, non-invasive analysis methods for measuring the diffusion of dCO_2_ in hydrogels are limited. In this article, we describe an indirect method for the non-invasive measurement of diffusion rates for dCO_2_ in hydrogels. We visually tracked the diffusion along the axial direction of pH indicator-doped hydrogel monoliths by recording the interface position over time. We calculated the interface velocity and the pseudo diffusion coefficients (D_pseudo_) over time. The obtained D_pseudo_ values are in a realistic range compared to literature values. Therefore, this novel analysis method for dCO_2_ diffusion gained valuable insights into diffusion dynamics in different hydrogels and can aid in the design of better immobilisation matrices for photosynthetic cells.

• Non-invasive, rapid method for estimation of dissolved CO_2_ (dCO_2_) diffusion in hydrogels
• Automatic analysis of colour interface formation due to acidification of hydrogels by diffusing dCO_2_
• Agarose hydrogels exhibit an approximated 30x higher pseudo dCO_2_ diffusion coefficient than silica gel

## Graphical abstract

**Figure:**
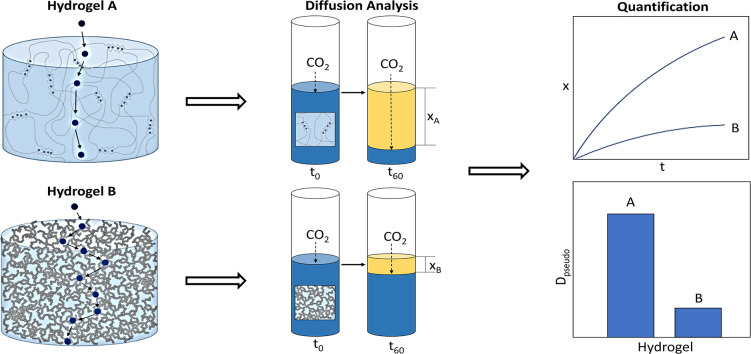

Specifications table

**Table:** 

Subject area:	Materials Science
More specific subject area:	Diffusion in Hydrogels
Name of your method:	Indicator-based Dye front Analysis (IDA)
Name and reference of original method:	None
Resource availability:	AnyCubic I3 Mega S with PLA (polyglycolic acid) Raspberry Pi 3 with a 12-megapixel wide-angle lens colour camera Open-source Python library (Python Software Foundation) Matlab (The MathWorks, Inc.) Open-source VNC Viewer software (Virtual Network Computing) Open-source WinSCP software (Windows Secure Copy) Microsoft Excel

## Background

Hydrogels can be produced from cross-linked natural and synthetic polymers which renders them capable of holding large amounts of water (70–90 %) in their three-dimensional network without losing their structural integrity [1,2]. This makes them attractive for biotechnological applications such as biosensors [3,4], delivery of bioactive agents [5,6], tissue engineering [7,8] or encapsulation of living cells [[9], [10], [11], [12], [13], [14]]. The encapsulation of living cells in hydrogels for industrial exploitation has generally been reported since 1964 [15]. Encapsulation offers numerous attractive features including high cell densities, ease of handling, a highly hydrated environment that protects from external influences like pH shifts, shear forces, UV light or contaminations, circumvention of the harvest problem as well as the facilitation of continuous processes by retaining valuable biomass [16]. This makes whole-cell encapsulation promising for producing highly valuable substances with industrial applications and health benefits [17]. Diverse hydrogels have been successfully applied as encapsulating matrices for mammalian cells [9], bacteria [10], cyanobacteria [11], microalgae [13] or yeast [14] due to their unique physical, structural and mechanical properties. However, the most critical step is selecting an encapsulating material with appropriate features for the individual cell types. For sensitive cells the encapsulation via reversible thermal and ionic gelation is limited due to the high temperatures and concentration of ions during the gelation process. Encapsulation in non-reversible silica hydrogels remains challenging due to organic solvents, toxic by-products and mineral acids or bases for pH adjustment [16]. Especially for encapsulating photosynthetic cells like microalgae and cyanobacteria which grow by converting sunlight and CO_2_ into sugars the diffusion properties of dissolved CO_2_ (dCO_2_) must be considered [18].

General methods for the analysis of gaseous CO_2_ diffusion phenomena include theoretical development of mathematical models and molecular dynamics simulations [19,20], concentration measurements [21,22], microelectrodes [23], Raman spectroscopy [24], X-ray Computer-Assisted Tomography [25], Nuclear Magnetic Resonance (NMR) [26] or Pressure-decay [27]. However, dissolved CO_2_ is generally more challenging to measure than gaseous CO_2_ because it is difficult to distinguish CO_2_ (aq) ions from other ions [28]. Indicator dyes like thymol blue, bromothymol blue, bromocresol green or bromocresol purple have been widely applied for the tracking of dissolved CO_2_ diffusion in water or porous media. The tracking of the diffusion-induced colour change is often performed in a two-dimensional Hele-Shaw cell followed by image analysis of the hydrodynamic fingering patterns [[29], [30], [31], [32]]. Other methods include a microfluidic approach that visualises CO_2_ diffusion in water by quenching of a fluorescence indicator or the application of an indicator dye-doped film for the photo spectroscopic analysis of CO_2_ diffusion in water [33,34]. However, none of these methods were suitable for the analysis of CO_2_ diffusion in hydrogels.

Thus, we developed the *Indicator-based Dye front Analysis* (IDA) method, which enables quick, simple, inexpensive, and non-destructive analysis of dCO_2_ diffusion in different hydrogels. The bromothymol blue-doped hydrogels are placed in glass vials and gassed with CO_2_ continuously. Since the resulting colour change appears as a straight line instead of hydrodynamic fingering, pseudo diffusion coefficients can be calculated from the taken images.

## Method details

### Materials, fabrication and procedure

**Fig. 1:**
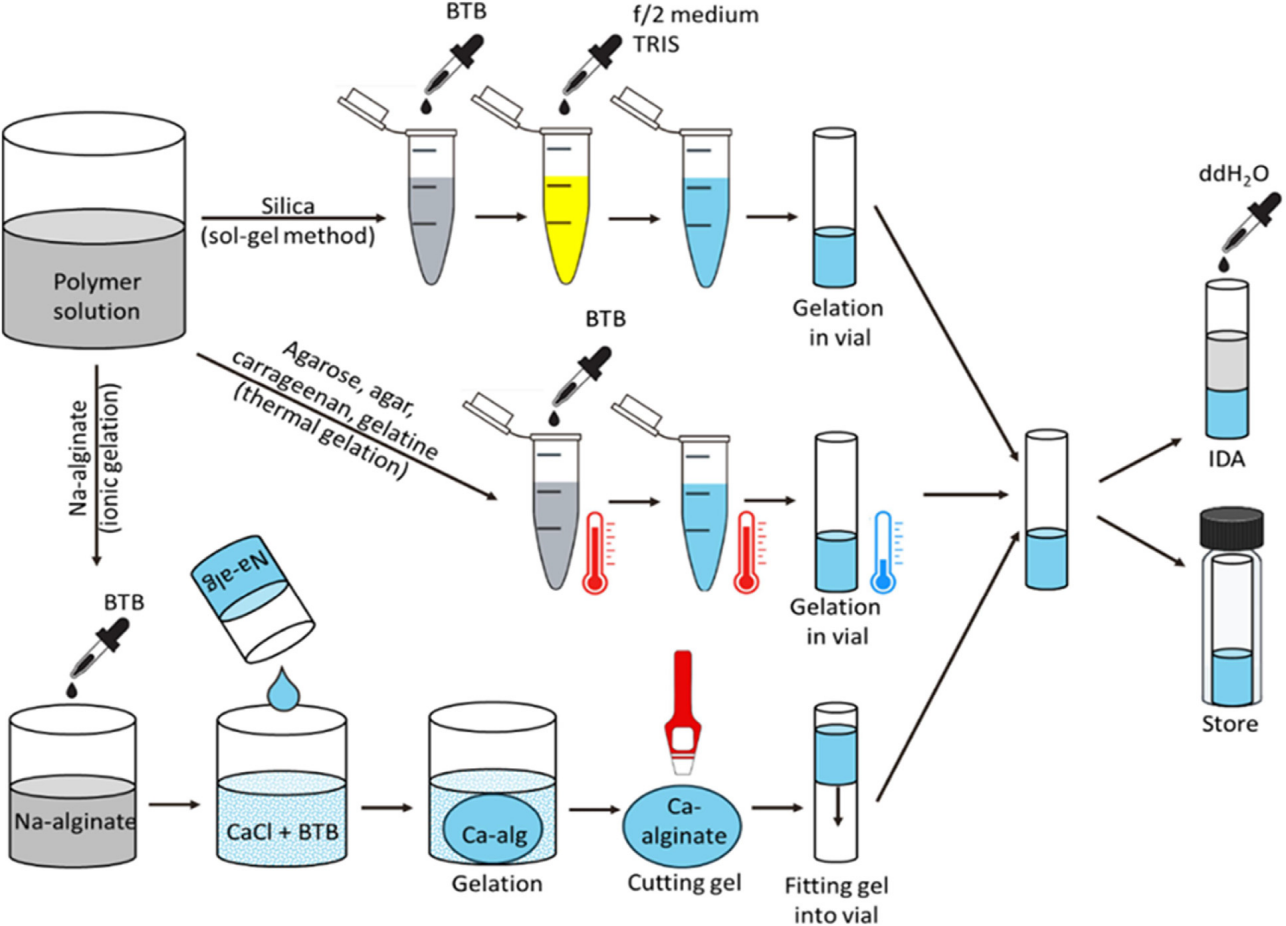
Sample preparation for the different hydrogels. Dye-doped bromothymol blue (BTB) silica hydrogels were obtained by the sol-gel method while the thermally gelling polymers were heated and gelled by cooling directly in the sample vials. The dye-doped Na-alginate was gelled in CaCl_2_ solution and the hardened Ca-alginate was cut to fit tightly into the sample tubes. All samples were either directly measured or stored in air-tight screw cap bottles at room temperature.

For a 10 % v/v sodium silicate sol, 9 mL distilled water, 1 mL sodium trisilicate solution (Na_2_O_7_Si_3_, 26.5 % SiO_2_) and 3.2 g protonated ion exchanger Amberlite IR-120 were stirred at 500 rpm for 15 min. The ion exchanger was removed via filtration and the sol was distributed into 1.5 mL aliquots in 2 mL reaction tubes. 75 µL 0.1 % aqueous bromothymol blue pH indicator solution and 300 µL of f/2 marine microalgae cultivation medium [35] were added to each aliquot (Fig. 1, top row).

Due to the acidic pH, the sol was yellow after this step. Bromothymol blue was chosen as an indicator for the analysis of hydrogels at a physiological pH of 8.0 since its deprotonated form had a deep blue colour at the beginning of the analysis, ensuring a high contrast to the yellow colour shift upon protonation. Gelation of the sol was initiated individually for each aliquot by adding 18 µL of 2 M tris(hydroxymethyl)-aminomethane (TRIS) and mixing thoroughly, which resulted in a colour change from yellow to blue at a final pH of 8.0. Before the gelation was completed, 150 µL of the blue activated sol were transferred into each of ten glass reaction vials, respectively, making for a complete measurement set.

Dye-doped agar, agarose, carrageenan and gelatin hydrogels were prepared by heating 1.818 mL of the respective polymer solution and adding 75 µL 0.1 % aqueous bromothymol blue pH indicator solution. The pH was adjusted to 8.0 with either 0.1 M NaOH or HCl and 150 µL of the hot mixture were filled into the vials, which were then left to harden for 5–10 mins at room temperature, except for gelatin, which was hardened at 5 °C overnight (Fig. 1, middle row). For calcium alginate-based gels, a large volume of sodium alginate solution was gelled in a beaker in 2 % dye-doped CaCl_2_ crosslinking solution for 48 h, resulting in a big hydrogel sphere. After complete gelation of the hydrogel sphere, cylinders were cut out with a 6 mm diameter cutting tool to obtain snug-fitting hydrogel cylinders to be inserted into the reaction vials (Fig. 1, bottom row). A thin syringe tip must be inserted into the vials before sliding in the cut gel to allow the air to escape. The slightly oversized hydrogel cylinder will afterwards fill the tiny gap between the gel and the vial wall after removing the syringe tip. This procedure is necessary for hydrogels that decrease their volume during gelation since there must be no gaps between the hydrogel and the walls of the vial. Any gaps would lead to a short circuit current of the gas around the hydrogels and improper dye front generation. All hydrogel vials were either stored in air-tight screw cap bottles at room temperature or analysed immediately. Before the analysis 200 µL MilliQ water were added to each sample vial to enable CO_2_ dissolution.

### Measuring principle

The measuring principle of IDA is based on the pH-dependent colour change of the pH indicator dye bromothymol blue. The dye is green at neutral pH, changing to yellow under acidic conditions and blue under alkaline conditions. It offers the best contrast between the alkaline and acidic colour in the required field between pH 6 and 8 and thus was preferred over other potential pH dyes (Table 1).

**Table 1:** pH indicators with their ideal pH range and resulting colours [36].

Indicator dye	Ideal pH range	Acid colour	Base colour
Thymol Blue	1.2–2.8	Red	Blue
Congo Red	3.0–5.0	Blue	Red
Methyl Orange	3.0–6.3	Red	Yellow
Bromocresol Green	4.0–5.6	Yellow	Blue
Methyl Red	4.2–6.2	Pink	Yellow
Bromocresol Purple	5.2–6.8	Yellow	Purple
Bromothymol Blue	6.0–7.6	Yellow	Blue
Neutral Red	6.8–8.0	Red	Yellow
Phenol Red	6.8–8.2	Yellow	Red
Phenolphthalein	8.0–10.0	Colourless	Pink
Thymolphthalein	8.8–10.5	Colourless	Blue

When CO_2_ is absorbed by water, a series of chemical reactions occur, resulting in increased hydrogen concentration and a pH lowering. The dissolution of gaseous CO_2_ (CO_2(gas)_) in water (Eq. (1)) results in the formation of aqueous CO_2_ (CO_2(aq)_) which rapidly reacts to form carbonic acid (H_2_CO_3_) as shown in Eq. (2). This weak unstable acid almost immediately dissociates into hydrogen ions (*H*^+^) and bicarbonate ions (HCO_3_^-^) as shown in Eq. (3), known as the first dissociation step lowering pH and changing indicator dye colour.

(1) $$CO_{2\left(\text{gas}\right)}↔\;\left[CO_{2\left(\text{aq}\right)}\right]$$

(2) $$\left[CO_{2\left(\text{aq}\right)}\right]+\;H_{2}O\;↔\;\left[H_{2}CO_{3\left(\text{aq}\right)}\right]$$

(3) $$\left[H_{2}CO_{3\left(\text{aq}\right)}\right]↔\;\left[H_{\left(\text{aq}\right)}^{+}\right]+\;\left[{\text{HCO}}_{3\left(\text{aq}\right)}^{-}\right]$$

(4) $$\left[{\text{HCO}}_{3\left(\text{aq}\right)}^{-}\right]\;↔\;\left[H_{\left(\text{aq}\right)}^{+}\right]+\;\left[{\text{CO}}_{3\left(\text{aq}\right)}^{2-}\right]$$

Most of the pH changes observed in testing come from the first equivalent dissociation. The second dissociation step (Eq. (4)) has a much smaller equilibrium constant and could be neglected without incurring significant errors [33].

**Fig. 2:**
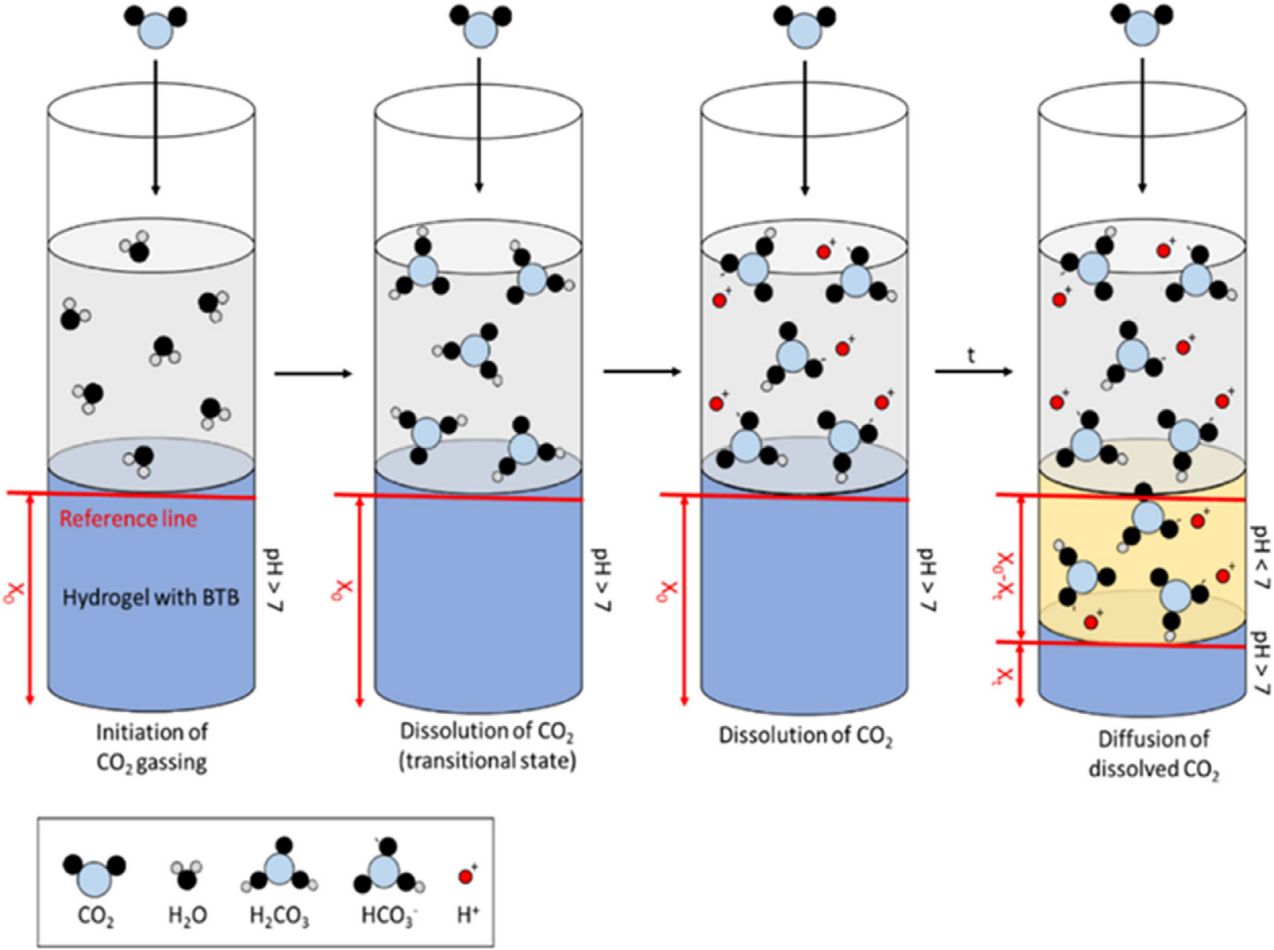
Measuring principle of the IDA method. The dye-doped blue hydrogel in the sample vial is covered by water. The first picture was taken without CO_2_ gassing so the line detection program could detect the reference and fix the lines. Dissolution and diffusion of CO_2_ into the hydrogel under gassing conditions leads to a decreased gel pH and thus a colour change to yellow with the formation of an interface between the colours. Over time the interface moves downwards as the diffusion of dissolved CO_2_ into the gel continues. The line-detecting program analyses the movement of the interface and aids in calculating diffusion parameters.

The deprotonated pH-indicating dye will capture the dissociated hydrogen ions (*H*^+^) and thus convert into the protonated form, resulting in a colour shift. Indicator-doped hydrogels were prepared around a physiological pH of 8.0 to create a high contrast between the blue colour at the beginning and the yellow colour upon dCO_2_ diffusion. As a result of constant CO_2_ gassing at 1 L/min, a yellow dye front is formed in the gel, which expands in correlation to the diffusion rate of the dissolved CO_2_ (Fig. 2).

### Design of the IDA measuring device

**Fig. 3:**
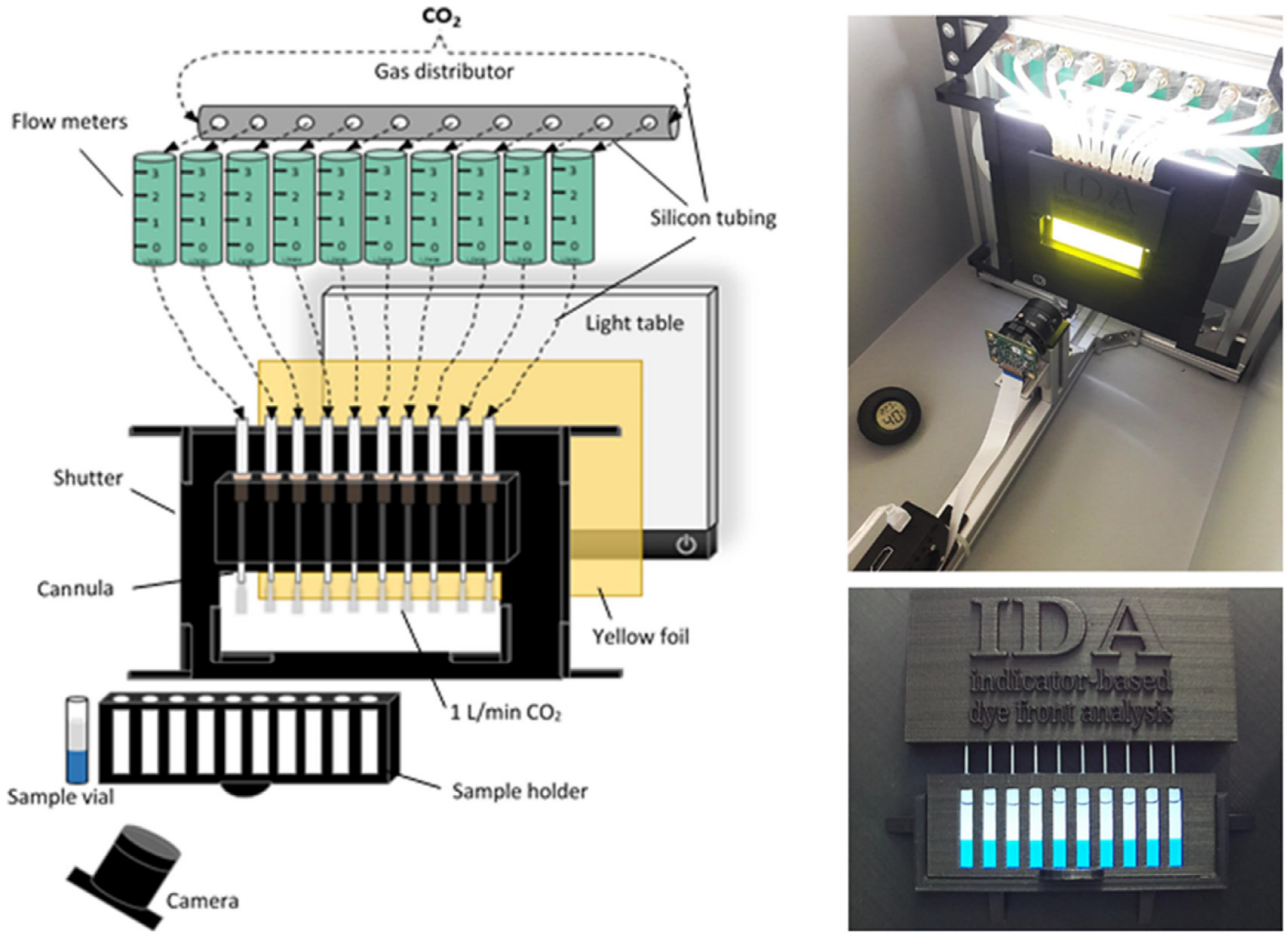
Laboratory setup of the IDA measuring device. The gaseous CO_2_ is distributed into ten individual flow meters by silicon tubing. With a flow rate of 1 L/min, it is directed into blunt cannulas that ensure even gassing of the ten gel-filled sample vials held by the sample holder and placed onto the shutter. A light table with a yellow filter foil illuminates the vials from the back to remove the green transition colour and thus reduce the number of detectable lines. A real-time camera takes pictures of the colour transitions of the gels over time.

All holding parts for the device were 3D printed, resulting in a perfect fit with cheap, fast and highly customisable construction (Fig. 3). The printing material PGA (polyglycolic acid) is durable, lab-safe and environmentally friendly. The sample holder for ten vials was designed for an optimal sample display, illumination, image capture and handling. For illumination, an LED table with neutral white light was coated with a milky polymer film to diffuse the light from the individual LED pixels increasing the scattering and thus homogenizing the light. A cost-efficient, yet powerful 12-megapixel Raspberry Pi 3 colour camera with a wide-angle lens and a focal length of 6 mm was chosen for real-time image capturing. The camera was controlled through an open-source Python library and is easy to use, small, modular and mobile. A light-blocking box covered the device to prevent the surrounding light from influencing the image capture. The gas flow of 1 L/min was controlled by ten individual flow meters with a range of 0–3 L/min for each sample tube. Standardised blunt cannulas with a diameter of 2 mm and a length of 40 mm were installed in a fitted holder for the uniform gas flow into the sample tubes. A detailed depiction of the IDA components as well as a step-by-step depiction of the measuring process can be found in the supplementary data (figure S1 and S2).

### Image capturing and automatic dye front identification

**Fig. 4:**
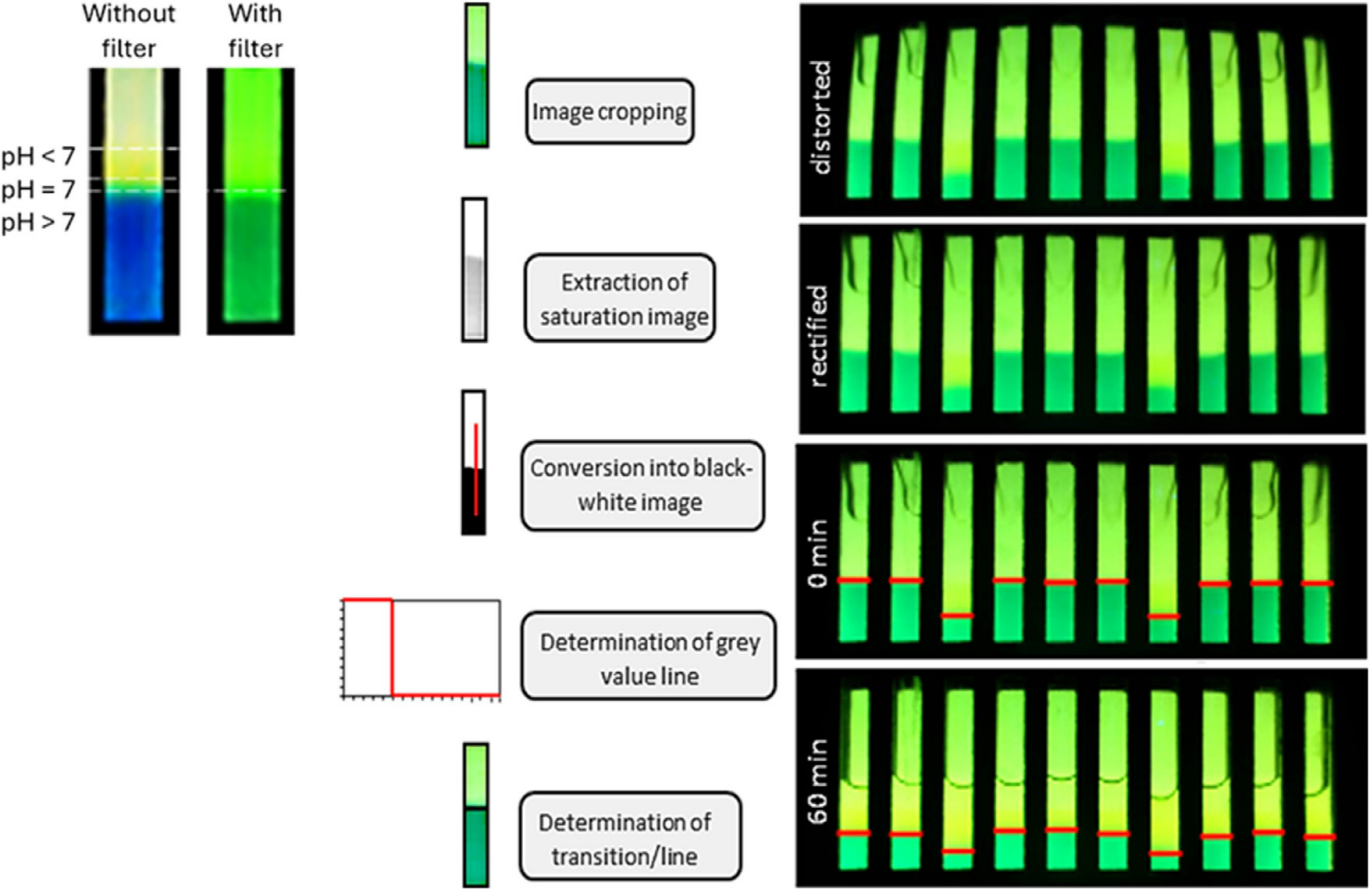
Left: Comparison of colour transition without and with a yellow colour filter film added to the light table. The filter merges the green transition colour with the background and thus reduces the number of visible transitions of the indicator. Right: The line detection program rectifies the raw pictures, and the individual vials are cropped. After the extraction of saturation images and conversion into black and white photos, grey value lines are determined for each vial. The transition lines between the yellow and blue parts of the hydrogels are defined and highlighted as red lines in the images. Values for the interface positions over time are extracted into an Excel file for further calculations.

The dye front detection was accomplished in the commercial software Matlab (The MathWorks, Inc.), which was installed on the user computer. The Raspberry Pi Camera was adjusted with the “Camera Calibrator” toolbox within Matlab. The camera is physically controlled by the Raspberry Pi 3 computer, which runs a Python script that provides a control surface for capturing and saving the images. The open-access VNC Viewer software (Virtual Network Computing) enables remote access to the Raspberry Pion the user computer with the Matlab software. Since the pH indicator does not show a binary change of colour from blue to yellow but rather a gradient from blue over green to yellow, a yellow colour filter film was added to the light table to reduce the number of visible transitions of the indicator. The final captured image contains only one visible interface of the gel, since the yellow and green colour of the gel merge with the background (Fig. 4, left). The blue colour of the gel has a much lower proportion of yellow and appears green in the captured image. Pictures of the moving interface were taken every five minutes over 60 mins. For the interface detection, the images were rectified to reduce the distortion by the camera lens (Fig. 4, right). Pictures were subsequently analysed with a line detection programme in Matlab that visualised the detected lines in red. A conversion factor of 0.0681 (6.81 mm displayed with 100 pixels) was determined and used for the calculations. After the measurement was finished the interface positions at every measured time point were exported as an Excel table for further calculations.

### Calculation of interface velocity, pseudo diffusion coefficients and porosity

The interface velocity (V_i_) in m/s was calculated by Eq. (5) and the pseudo diffusion coefficient (D_pseudo_) in m^2^/s was calculated by Eq. (6) based on the Stokes-Einstein law [37] for each time spot:

(5) $$V_{i}=\;\frac{x_{0}-\;x_{t}}{t}$$

(6) $$D_{\text{pseudo}}=\frac{{\left(x_{0\;}-\;x_{t}\right)}^{2}}{2t}$$

Where x_0_ is the initial interface position and x_t_ is the position of the interface at time t.

The porosity of the hydrogels (θ) was calculated from Eq. (7) by weighing the wet and dry gels:

(7) $$\theta =\;\frac{V_{t}\;-\;V_{b}}{V_{t\;}}$$

Where V_t_ is the total weight of the wet hydrogel and V_b_ is the weight of the particular bulk material which is equal to the weight of the hydrogel after drying.

## Method validation

### Visualisation of diffusion behaviour in hydrogels

**Fig. 5:**
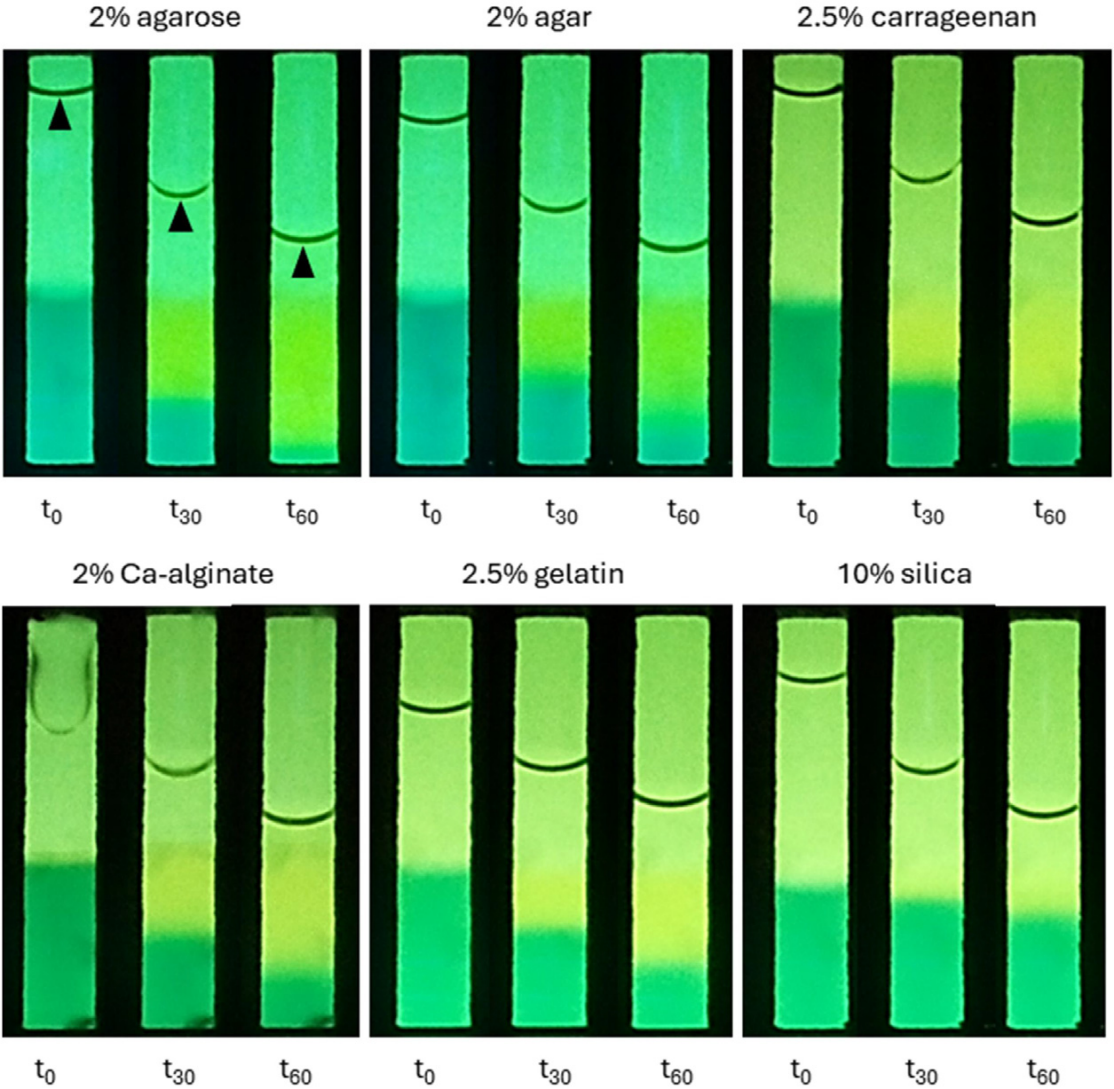
Visualisation of dissolved CO_2_ diffusion and migration of the colour interface in the different hydrogels at the beginning (t_0_), after 30 mins (t_30_) and at the end (t_60_). The black arrows indicate the water's evaporation over time and thus the lowering of the meniscus. However, the gels were not running dry at any point in time and the water line did not interfere with the line detection software. When compared, the difference of the interface position during the first 30 mins is more significant than that of the second 30 mins for all gels, which indicates a diffusion limitation over time and increasing diffusion distance. It is visible that the hydrogels differ in their diffusion properties, as the agarose gel has almost completely turned yellow after 60 mins. Agar, Ca-alginate and carrageenan gels show comparable interface positions, whereas gelatin has a higher and silica has the highest interface position after 60 mins.

At the beginning of the CO_2_ injection, a fast change of colour in the agarose hydrogels indicated that the CO_2_ dissolution in water and diffusion into the gel matrix occurs within seconds after initiation of gas injection. The formation of a dye front in the agar, Ca-alginate, carrageenan, gelatin and silica hydrogels became visible more slowly despite the immediate gas dissolution in water due to the lower diffusivity of these gels. The diffusion behaviour of the dissolved CO_2_ in the hydrogels was tracked in five-minute intervals for an hour and the differences of the interface position at the beginning (t_0_), after 30 mins (t_30_) and 60 mins (t_60_) are shown in Fig. 5. All interfaces were moving downwards in a straight line and no leakage of the CO_2_ along the sides of the vials was observed which would have been visible by yellowing of the gel in those areas. As expected, the meniscus of the water was moving downwards as well since the evaporated water was not replaced during the measurement (Fig. 5, black arrows). A video showing the dCO_2_ diffusion in agar can be found in the supplementary data.

### Quantification of diffusion behaviour in hydrogels: purified biopolymer gel performs strongest

**Fig. 6:**
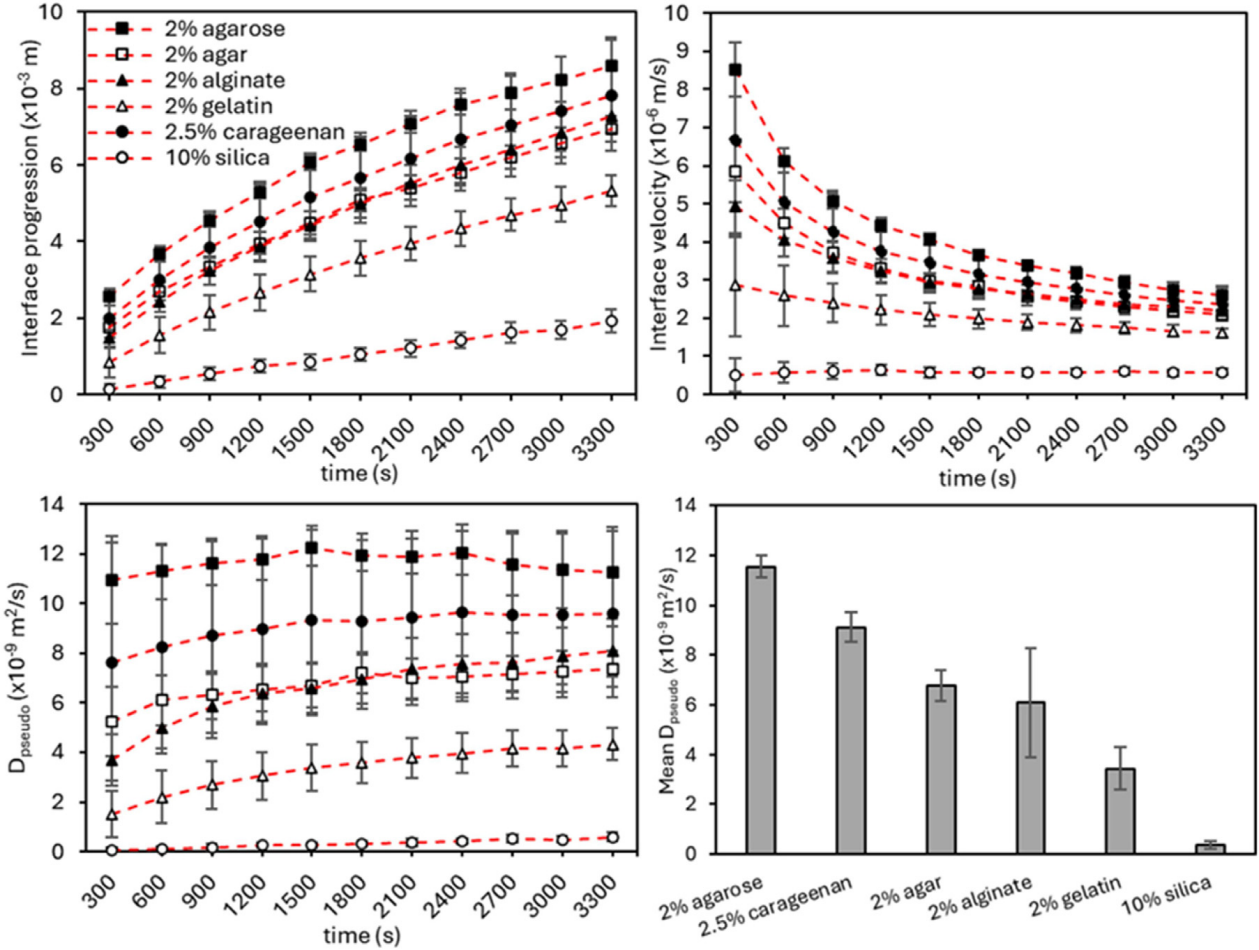
Dissolved CO_2_ diffusion behaviour of the tested hydrogels (standard deviation with *n* = 10).

We observed comparable patterns for the interface progression and velocity upon dCO_2_ diffusion in 2 % agarose, 2 % agar, 2 % Ca-alginate, 2 % gelatin, 2.5 % carrageenan and 10 % silica hydrogels. Across all samples the slopes are steeper in the first stages of the reaction (300–1200 s) than that during the later stages (1500–3300 s) (Fig. 6). The same phenomenon was previously reported in a study about the visualization of one-dimensional diffusion in hydrogels and the authors explained the decline by diminishing concentration differences as the diffusion continues over time [38].

Notably, there are performance differences between the different hydrogels, with the polysaccharide biopolymer agarose showing the highest and the inorganic silica the slowest dCO_2_ diffusivity (Fig. 6). This can be explained by the fact that the used electrophoresis-grade agarose was purified from the non-gelling component agaropectin, which contains a significant amount of negatively charged sulphate and carboxyl groups. Thus, it contains fewer charged groups than crude agar. Moreover, the adsorption of biomolecules that might interfere with the dCO_2_ diffusion is reduced. The other polysaccharide biopolymers Ca-alginate and carrageenan show comparable pseudo diffusion to that of agarose and agar since their molecular structures are similar. The gelatin hydrogels show a lower D_pseudo_, which might be due to the proteinaceous nature of this polymer. The weak performance and the over 30-fold decreased pseudo diffusion coefficient of the inorganic silica hydrogel compared with agarose can be explained by the lack of long, flexible polymer or protein chains and thus a much denser structure.

**Fig. 7:**
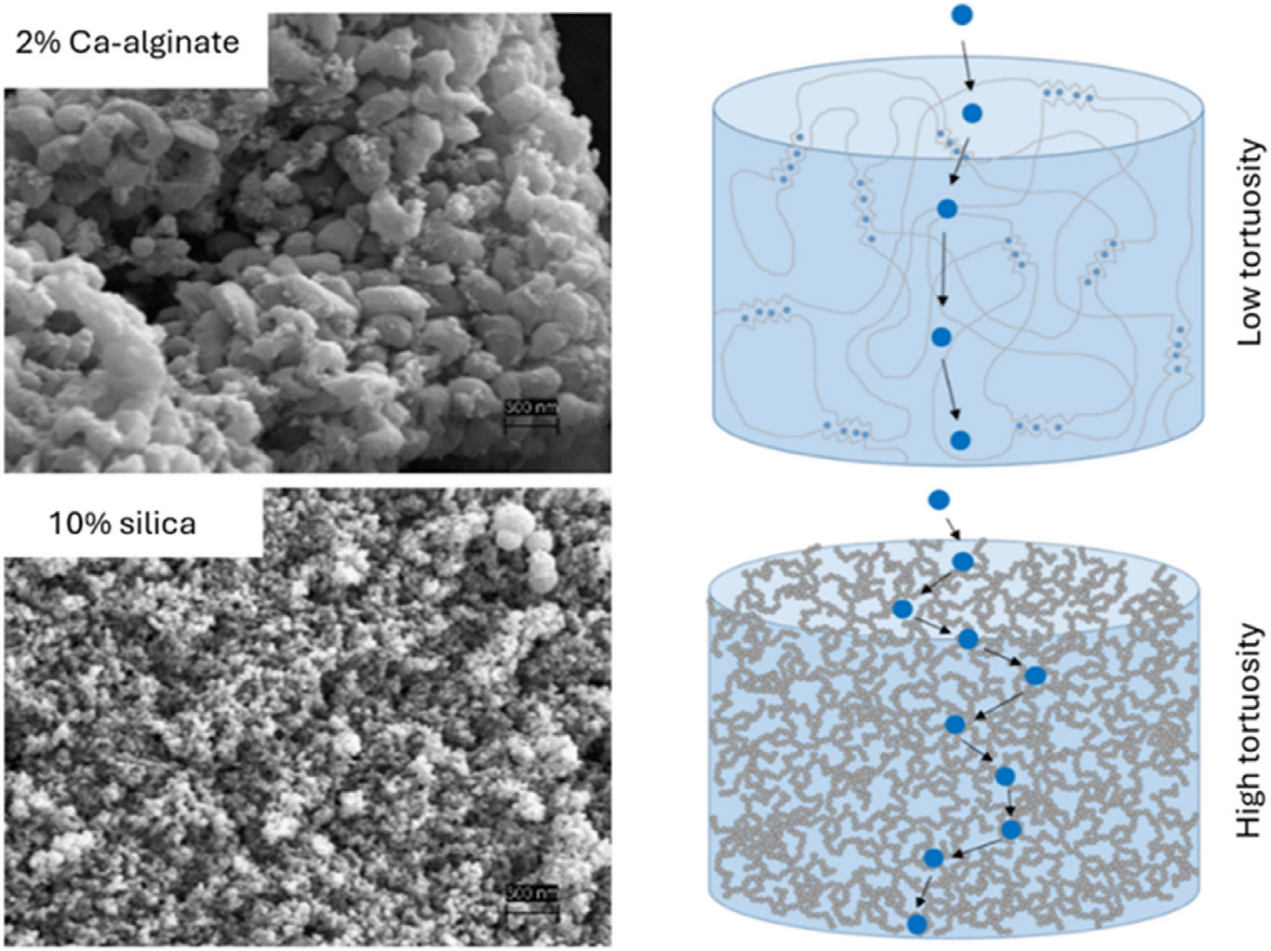
SEM images of Ca-alginate and silica hydrogels and visualisation of tortuosity effects on particle diffusion (scale bar: 500 nm).

To prove the assumptions about the silica hydrogels, the structure of Ca-alginate, the most commonly used hydrogels for living cell encapsulation, and silica were analysed by REM. The images highlighted the differences in pore size and proved a much denser structure and smaller pores in silica hydrogels (Fig. 7).

Additionally, the porosity (θ) was determined by weighing the wet and dry gels. Since θ values for Ca-alginate and silica are comparable with approximately 0.958 ± 0.006 and 0.956 ± 0.10, respectively, an increased tortuosity (τ) seems likely to be the reason for the reduced dCO_2_ pseudo diffusion coefficient in the silica gels. The high number of negative charges in sol-gels might also interfere with the diffusion.

The reported pseudo diffusion coefficients for dissolved CO_2_ in water, brine, saline, mucin, oil or porous media measured with conventional methods range from 0.47 to 12.21 × 10^–9^ m^2^/s depending on temperature, pressure and medium but mainly circle around 2 × 10^–9^ m^2^/s [[19], [20], [21], [22],[24], [25], [26], [27],^31^,^33^,^39^]. Our IDA results range from 0.34 to 11.55 × 10^–9^ m^2^/s at room temperature, a gas pressure of 0.4 bar and a gas flow rate of 1 L/min depending on the hydrogel (Table 2). Notably, the scope of the present work was not to deliver absolute D_eff_ values for dissolved CO_2_ diffusion but to provide estimations for comparing samples measured by the IDA method.

**Table 2:** Pseudo diffusion coefficient (D_pseudo_) for the tested hydrogels (standard deviation with *n* = 10).

Hydrogel	Mean D_pseudo_ (x 10^–9^ m^2^/s)
2.0 % agarose	11.55 ± 0.42
2.0 % agar	6.78 ± 0.61
2.0 % Ca-alginate	6.07 ± 2.20
2.5 % carrageenan	9.11 ± 0.59
2.0 % gelatin	3.42 ± 0.86
10.0 % silica	0.34 ± 0.17

Concentration-Dependent Diffusion in Hydrogels: Compliance to Fick's First Law of Diffusion

**Fig. 8:**
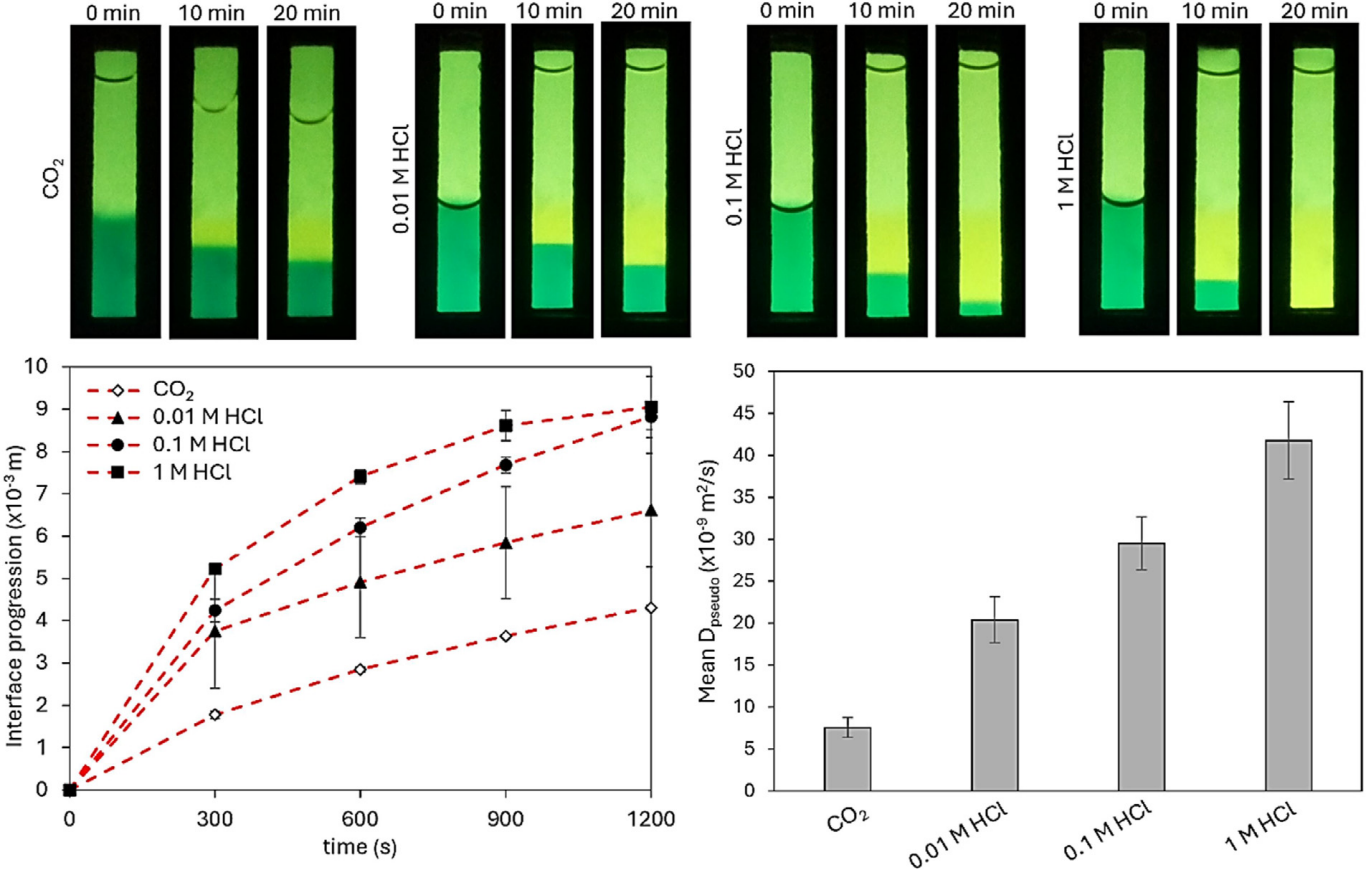
CO_2_ and HCl diffusion behaviour in 0.5 % agar (standard deviation with *n* = 5).

Fick's first law of diffusion states that the movement of particles from high to low concentration is directly proportional to the particle's concentration gradient [40]. To roughly demonstrate compliance with Fick's law the diffusion of different concentrations of HCl in 0.5 % agar was analysed since the IDA device did not allow for a sufficient increase of CO_2_ pressure. As shown in Fig. 8 the interface progression as well as the pseudo diffusion coefficients increase with increased HCl concentration. This is in accordance with a previous study where the authors demonstrated that Fick's law can be visualized by one-directional diffusion in hydrogels as the diffusion was faster at higher reagent concentrations [38].

In summary, the IDA method is a functioning visualisation tool for analysing dissolved CO_2_ diffusion in hydrogels. The ability of this method to detect discrepancies in the diffusion of dCO_2_ in different hydrogels makes it a valuable tool for the investigation of hydrogel behaviour under microalgae cultivation conditions. It is essential to investigate the diffusion of this gas specifically since microalgae cultivation is often performed with CO_2_ as a carbon source. Compared to direct analysis methods which can be time-consuming, invasive, destructive, expensive and complicated to handle the IDA method enables comparative investigations in a time-efficient, non-invasive and reproducible manner. It yields the interface velocity and pseudo diffusion coefficients which range in the field described in literature (10^-9^m^2^/s) and can be interpreted comparatively. Application of IDA measurements under cultivation conditions can aid in optimising hydrogel properties to avoid CO_2_ limitations and enable optimal growth of encapsulated photoautotrophic microalgae. This provides a valuable tool not only for making encapsulated phototrophic microalgae cultivation more feasible in the future but also for better understanding diffusion phenomena in hydrogels in general. With the help of suitable indicators, the diffusion of other important molecules like oxygen or nitrate could also be investigated with this setup.

## Limitations

**Fig. 9:**
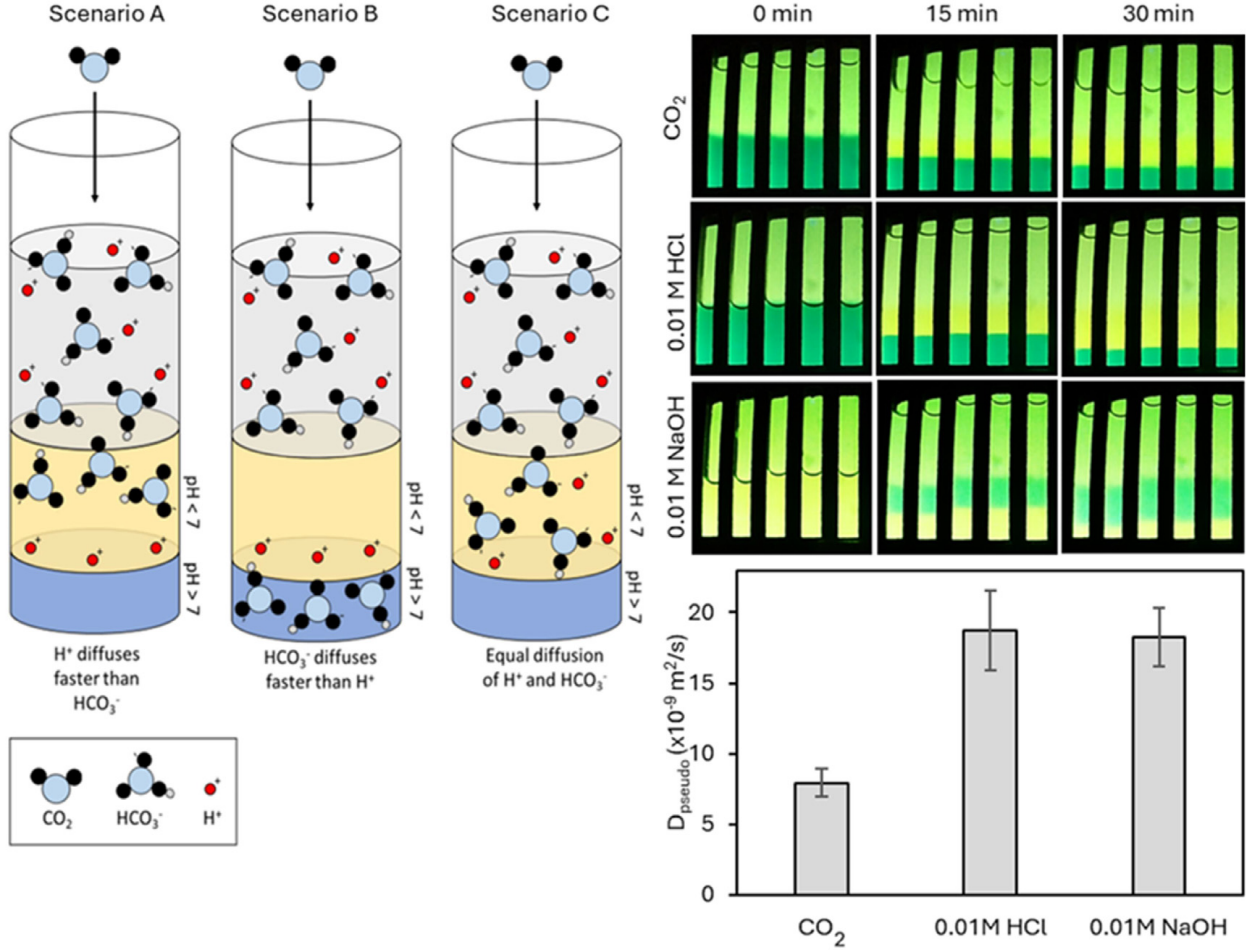
Comparison of acid and base diffusion in 0.5 % agar with dCO_2_ diffusion as a reference (standard deviation with *n* = 10). Scenarios A, B and C illustrate potential diffusion behaviours upon diffusion of dissolved CO_2_ that result in the same colour change of the indicator and thus can only be assumed. Scenario A shows a faster diffusion of protons than bicarbonate ions, scenario B illustrates the opposite behaviour and scenario C depicts an equally fast diffusion of both moieties.

The IDA analysis is an indirect method to investigate dCO_2_ diffusion in hydrogels since it tracks the diffusion of *H*^+^ that develops during the dissolution of CO_2_ into *H*^+^ and HCO_3_^-^ in water. Those hydrogen ions can diffuse approximately seven times faster than other small ions in water due to the Grotthuss mechanism (D_H+_ = 9.3 × 10^–9^ m^2^/s) [41]. This could potentially lead to overestimation of the dCO_2_ diffusion in hydrogels. However, it is known that the diffusion of *H*^+^ in agarose hydrogels is limited due to structural interactions between the polymer chain and the diffusing ions and thus hindered proton hopping (D_H+_ = 6,2 × 10^–9^ m^2^/s) [42]. It is probable that the spatial arrangement of polymer chains interferes with the hopping protons and thus reduces the efficiency of the Grotthuss mechanism. In hydrogels containing counterions, the polyelectrolyte backbone can further hinder the diffusion of hydrated hydrogen ions [42]. A comparative measurement with HCl and NaOH was performed to investigate whether the diffusion of *H*^+^ is comparable to the diffusion of negatively charged moieties in the IDA setup. Therefore, the pseudo diffusion coefficients of 0.01 M HCl and NaOH in 0.5 % agar were determined. For NaOH diffusion the indicator dye was adjusted to a yellow starting colour (pH 6) since during OH^-^ diffusion the gel gets alkalized and changes colour to blue. Upon acid diffusion the *H*^+^ caused the dye to change from blue to yellow as it did for the dCO_2_ diffusion. The dye front of the base diffusion was analysed manually to determine D_pseudo_ as the automated Matlab program only detects a colour change from blue to yellow. The pseudo diffusion coefficients were higher than that of dCO_2_ but comparable for HCl and NaOH (Fig. 9, right). Additional measurements with different concentrations of HCl and NaOH as well as CH_3_COOH and Na_2_CO_3_ showed the same comparability between the diffusion of protons and negative moieties (supplementary data, figure S3). Thus it can be concluded that in the agar hydrogel measured with the IDA device the diffusion differences between *H*^+^ and negative moieties (also probably HCO_3_^-^ from dCO_2_) are not as pronounced as in water, probably due to the hindered proton hopping as stated by Schuszter et al. in 2017 [42].

Based on these results the pseudo diffusion coefficients for HCO_3_^-^ diffusion upon dCO_2_ diffusion can be viewed as similar to those of *H*^+^in our specific measurement setup. Thus, scenario C (Fig. 9, left) can be assumed for dCO_2_ diffusion in hydrogels measured with the IDA.

It is worth noticing that the simplified diffusion principles mentioned in this article are aimed to develop an approachable indirect measuring device. The whole process of diffusion in hydrogels is much more complex, involving transport of actuating species, reactions with network components, destabilization of physical crosslinks or cleavage of network strands and concomitant network relaxation [43]. Certain hydrogels might be able to change their extent of swelling or other properties depending on environmental factors such as temperature, light or pressure, or specific molecules. Thus, complex theoretical reaction-diffusion models considering changes in the crosslinking density, various geometries, constraints and swelling of the gels are highly valuable for the in-depth analysis of diffusion processes in hydrogels [43]. Furthermore, in-depth experimental analysis of hydrogel network parameters such as pore structures or crosslinking density may further justify the results obtained by the IDA analysis. Methods like electron microscopy, X-ray, determination of molecular weight, crosslink density, and size fraction, swelling analyses, rheological tests or determination of the dynamic modulus can be applied to thoroughly characterize a hydrogel [44].

In addition to our IDA data, those complex theoretical reaction-diffusion models and in-depth network parameter analyses can help to ensure the success of the gel performance.

## Credit author statement

Laura Fladung: Conceptualization, Data curation, Formal analysis, Investigation, Methodology, Validation, Writing - original draft, Visualization. Sarah Vanessa Langwald: Funding acquisition, Supervision, Project administration, Writing - review & editing. Olaf Kruse: Supervision, Writing - review & editing. Anant Patel: Funding acquisition, Supervision, Writing - review & editing, resources.

## Ethics statements

None.

## Declaration of competing interest

The authors declare that they have no known competing financial interests or personal relationships that could have appeared to influence the work reported in this paper.

## Data Availability

The data that has been used is confidential.

## Appendix Supplementary materials

Image, video 1

Image, application 2

Image, image 3
