# Supplementary material for: A novel indicator-based visualisation method to investigate diffusion behaviour of dissolved CO2 in hydrogels
Source: MethodsX. 2025 Feb 17;14:103225. doi: 10.1016/j.mex.2025.103225 (PMC11910120; doi:10.1016/j.mex.2025.103225)
Supplement: Supplementary file 2 [file mmc2.docx]

**Supplementary material *and/or* additional information [OPTIONAL]**

A video showing the diffusion process of dCO_2_ in an agar hydrogel is available.


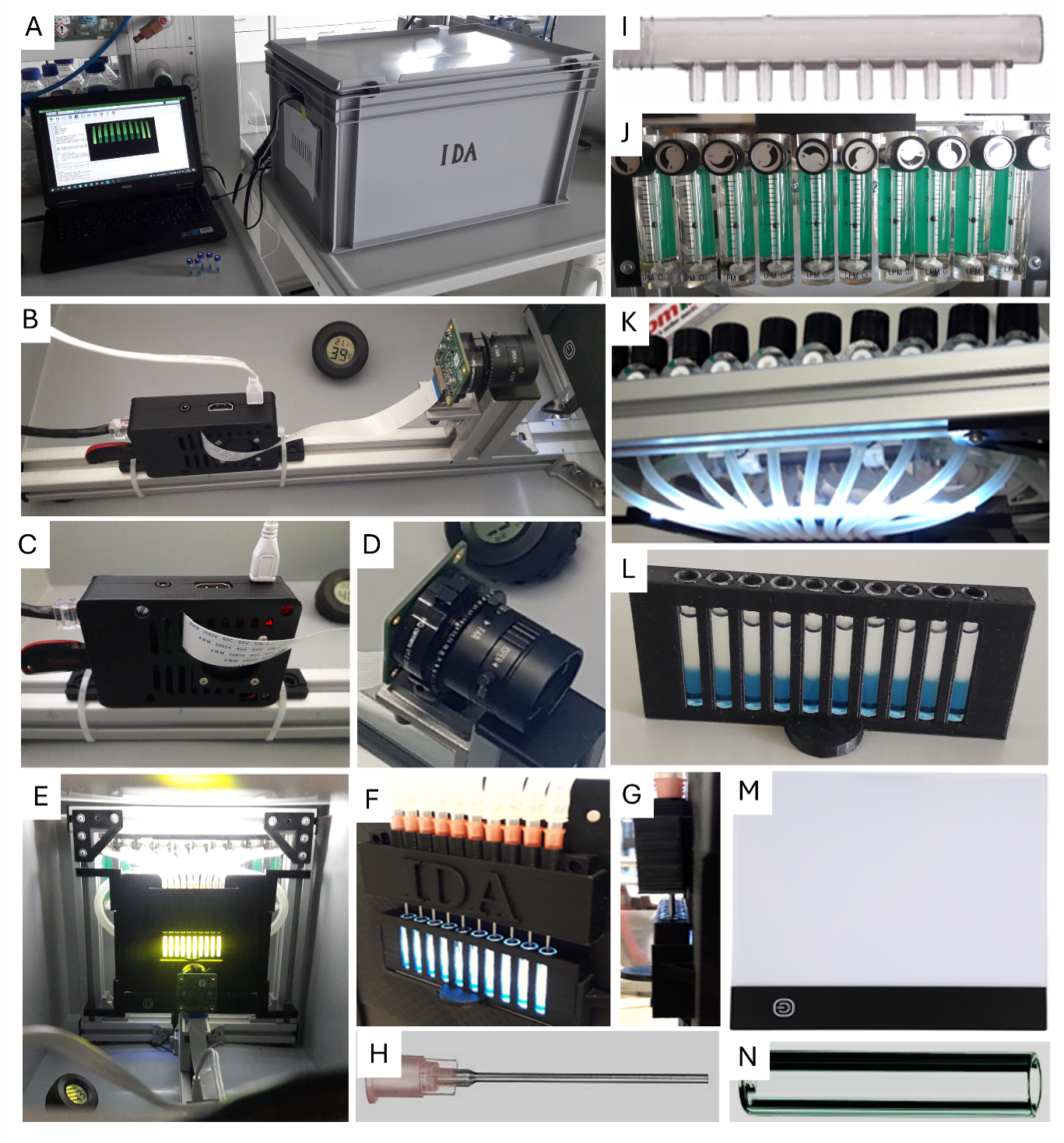


Figure S1: Components of the IDA device in detail: (A) IDA apparatus inside a light blocking box connected to a computer (B) Raspberry Pi 3 processor and camera adjustable on a 3.0 x 3.0 cm aluminum frame. Thermometer/hygrometer combination for temperature and humidity control (C) Raspberry Pi 3 processor controlled through an open-source *Python* library (D) 12-megapixel Raspberry Pi colour camera with a wide-angle lens and a focal length of 6 mm (E) Front view of the IDA device inside the light blocking box. The white light of the light table was changed to yellow with a filter foil for colour correction of the images. The base plate was 3D-printed with an AnyCubic I3 Mega S with 1.75 mm diameter PLA (polyglycolic acid). It is attached to a 3.0 x 3.0 cm aluminum frame (F) Close up of the base plate holding the ten cannula and sample holder with ten sample vials (G) Sideview of the cannula inside the sample vials (H) 2 mm x 40 mm blunt tip cannula (I) 12 cm x 1 cm gas distribution pipe with 10 outlets (J) Acrylic flow meters adjustable to 0-3 L/min (K) Top view of gas distribution to flow meters attached to a 3.0 x 3.0 cm aluminum frame (L) Sample holder with 10 hydrogel filled sample vials. 3D printed with an AnyCubic I3 Mega S with 1.75 mm diameter PLA (M) USB powered A5 LED light table with neutral white light and continuous brightness adjustment (N) 6 mm x 31 mm flat bottom glass vial (350 µl).


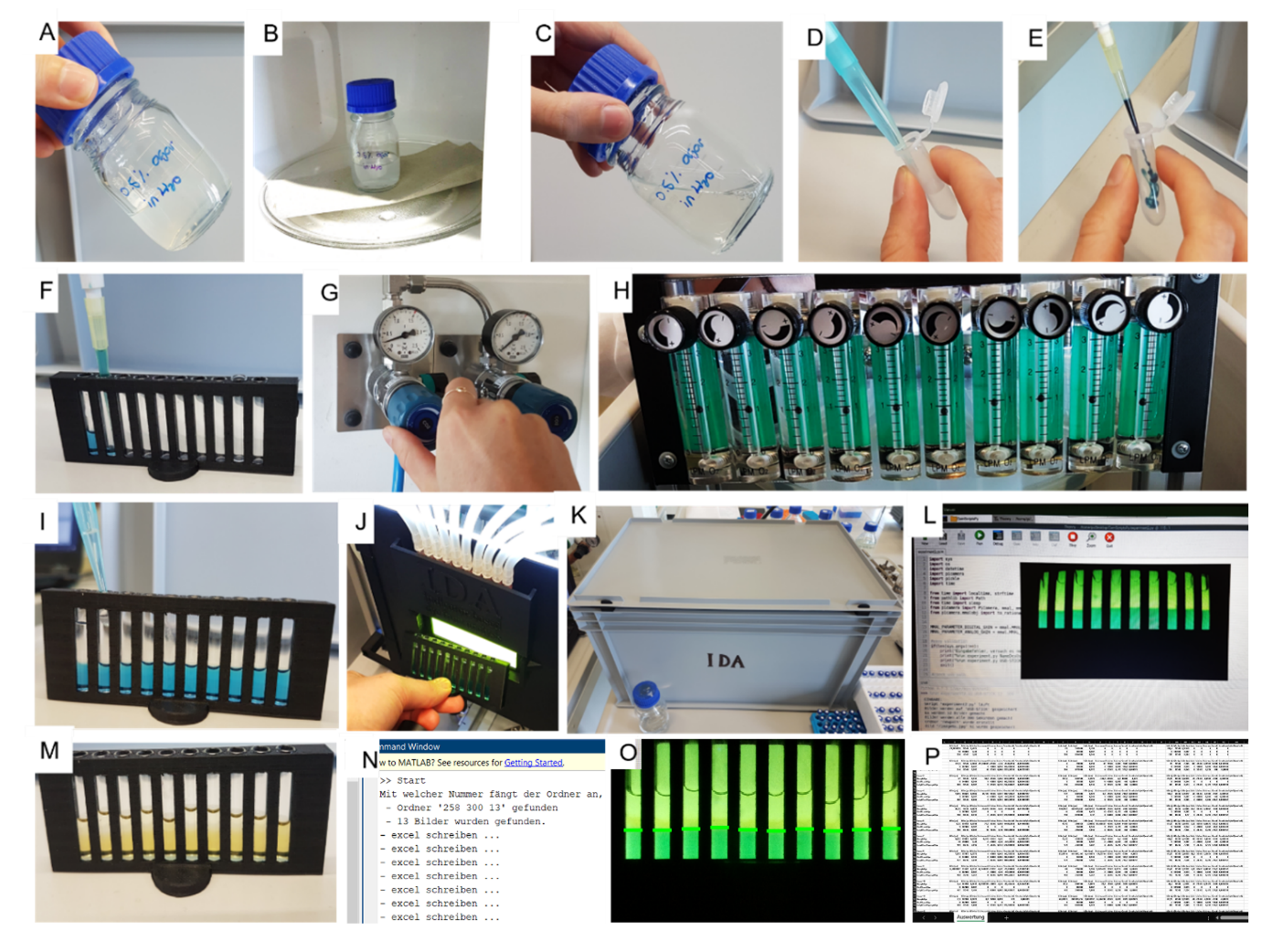


Figure S2: dCO_2_ diffusion measurement of an 0.5% agar hydrogel with the IDA device in detail. (A) Solid hydrogel in the storage bottle (B) Liquefication of the hydrogel by microwave heating (C) Liquid polymer solution ready for further processing (D) Transfer of 1.818 mL liquid polymer solution into a 2 mL reaction tube (E) Addition of 75 µL 0.1% bromothymol blue pH indicator into the polymer solution followed by thorough mixing (F) Transfer of 150 µL dye-doped polymer solution into each of the ten sample vials in the sample holder followed by subsequent gelation at room temperature (G) Adjusting the gas pressure to 0.4 bar on the pressure gauge (H) Adjusting each flow meter to 1 L/min (I) Addition of 200 µl MilliQ water to the top of each solid hydrogel in the sample vials (J) Insertion of the loaded sample holder into the IDA measuring apparatus (K) Closing the lid to prevent extraneous light (L) Initiation of the automatic image capture by starting the *Phyton* Script in the *VNC Viewer* software. Pictures of the ten samples are taken every 5 minutes over the period of one hour (M) After one hour of diffusion the hydrogels have changed colour from blue to yellow and can be removed from the device (N) Transfer of the images via the *WinSCP* software and automatic analysis of the dye fronts in *MatLab* (O) Indication of dye fronts by coloured lines for each image in *Matlab* (P) Automatic conversion of diffusion data from *Matlab* into a Microsoft Excel table for further analysis.


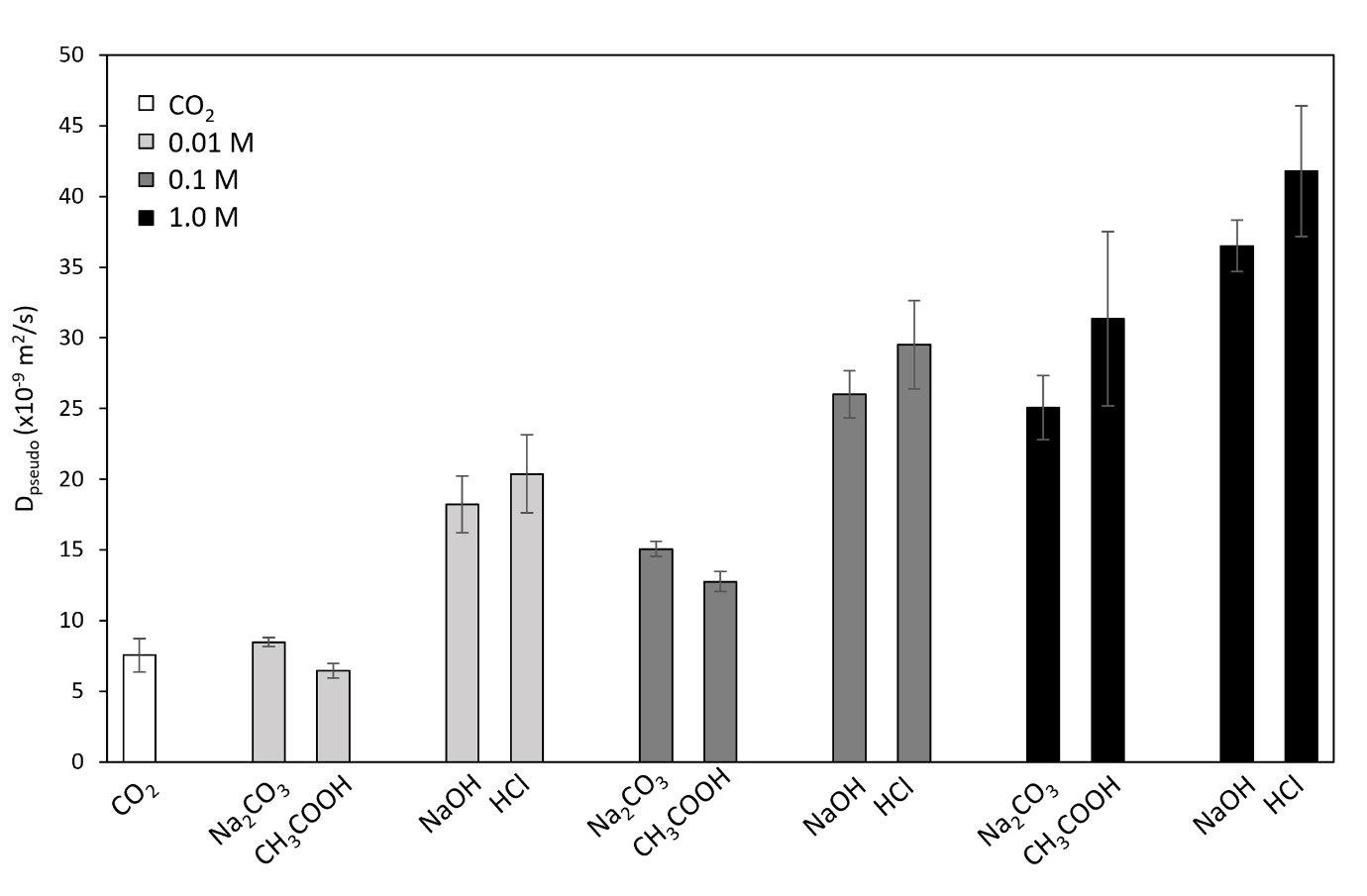


Figure S3: Pseudo diffusion coefficients for CO_2_, Na_2_CO_3_ and CH_3_COOH as well as NaOH and HCl at different concentrations in 0.5% agar hydrogels. The pseudo diffusion coefficients for the weak acid/base pair Na_2_CO_3/_CH_3_COOH and the strong pair HCl/NaOH are comparable, especially at lower concentrations (standard deviation with n=5).
