# Supplementary figures and images for: A novel indicator-based visualisation method to investigate diffusion behaviour of dissolved CO2 in hydrogels
Source: MethodsX. 2025 Feb 17;14:103225. doi: 10.1016/j.mex.2025.103225 (PMC11910120; doi:10.1016/j.mex.2025.103225)

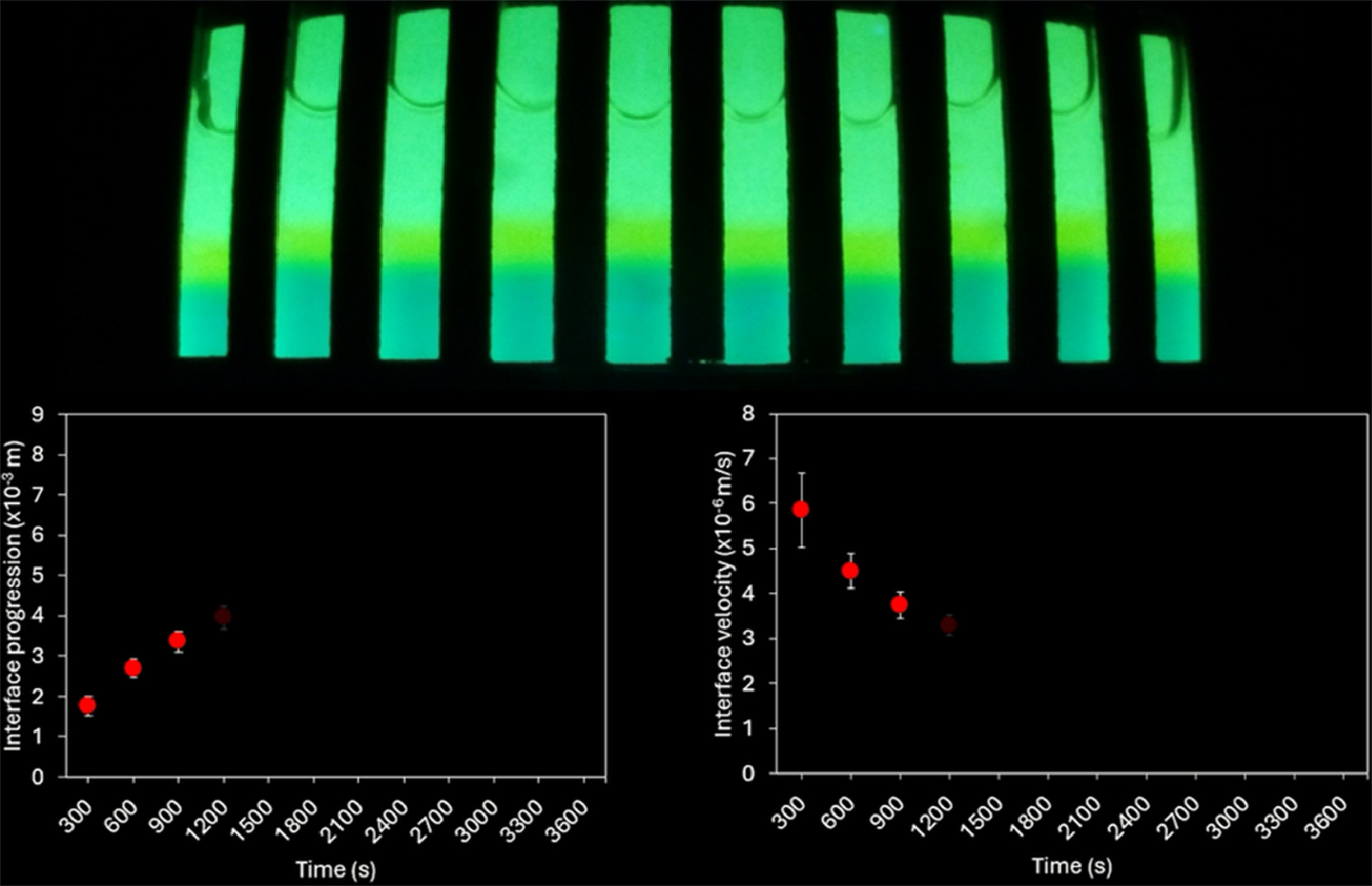

Supplement: Supplementary file 3 [file mmc3.jpg]
